# Supplementary figures and images for: Parasitism causes changes in caterpillar odours and associated bacterial communities with consequences for host-location by a hyperparasitoid
Source: PLoS Pathog. 2023 Mar 22;19(3):e1011262. doi: 10.1371/journal.ppat.1011262 (PMC10069771; doi:10.1371/journal.ppat.1011262)

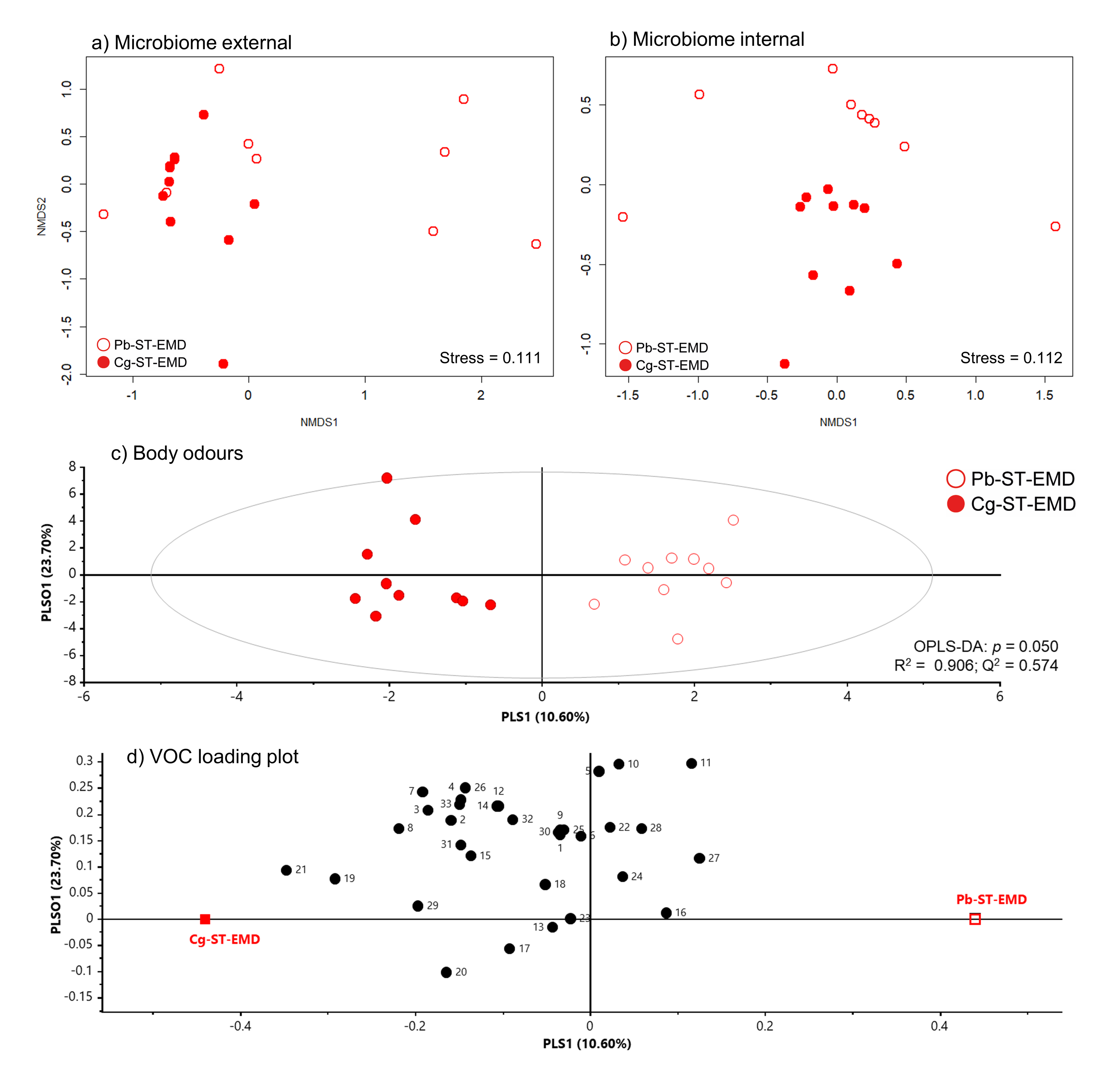

Supplement: S1 Fig — a) NMDS ordination plots based on Bray–Curtis distances of Hellinger-transformed relative abundance data of the external (dermal) bacterial communities. b) NMDS ordination for the internal bacterial communites. c) OPLS-DA plot for the volatile blends of different groups of caterpillars. The Hotelling’s T2 ellipse confines the confidence region (95%) of the score plot. d) The loading plot defining the contribution of each of the volatile compound to the separation of treatment groups. Volatiles compounds closer to a treatment in the plot are more correlated. For compound identity see Table 1. Abbreviations used: Cg-ST+EMD = starved then external microbiome disrupted parasitised caterpillars. Pb-ST+EMD = starved then external microbiome disrupted unparasitised caterpillars. (TIF) [file ppat.1011262.s001.tif]

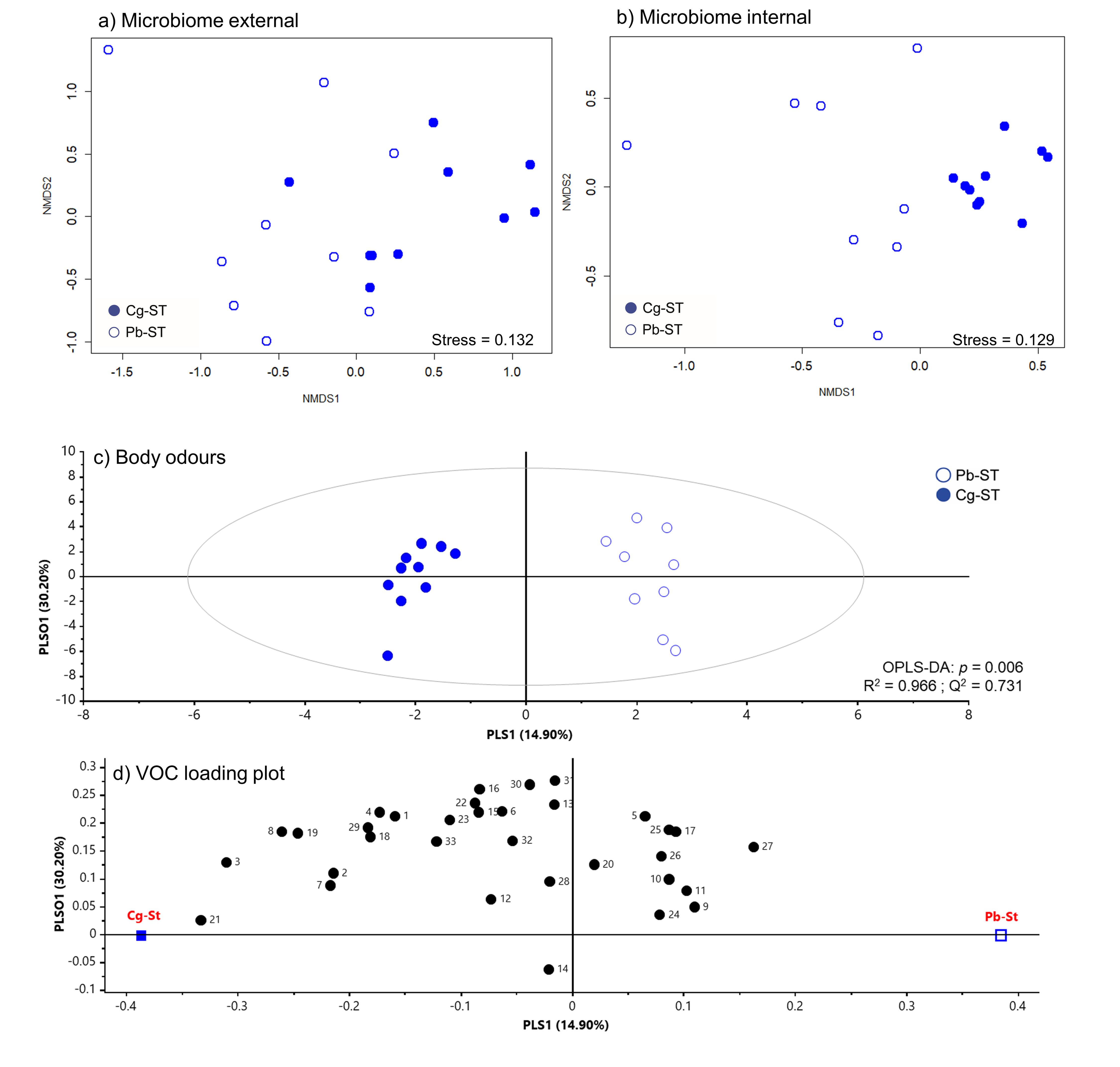

Supplement: S2 Fig — a) NMDS ordination plots based on Bray–Curtis distances of Hellinger-transformed relative abundance data of the external (dermal) bacterial communities. b) NMDS ordination for the internal bacterial communites. c) OPLS-DA plot for the volatile blends of different groups of caterpillars. The Hotelling’s T2 ellipse confines the confidence region (95%) of the score plot. d) The loading plot defining the contribution of each of the volatile compound to the orientation of sample groups in the score plot. Volatiles compounds closer to a treatment in the plot are more correlated. For compound identity see Table 1. Abbreviations used: Cg-ST = starved parasitised caterpillar. Pb-ST = starved unparasitised caterpillar. (TIF) [file ppat.1011262.s002.tif]

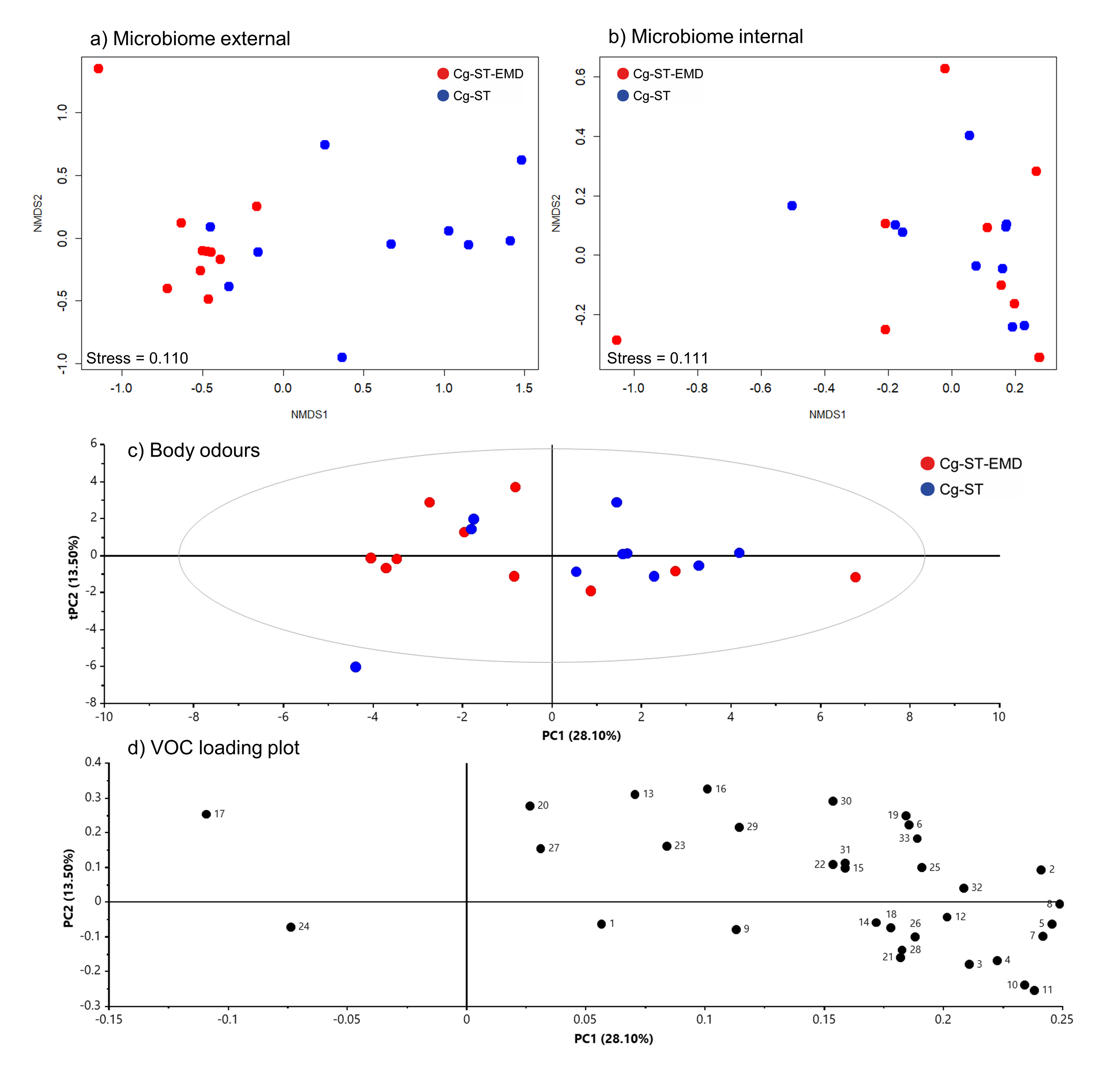

Supplement: S3 Fig — a) NMDS ordination plots based on Bray–Curtis distances of Hellinger-transformed relative abundance data of the external (dermal) bacterial communities. b) NMDS ordination for the internal bacterial communites. c) PCA plot for the volatile blends of different groups of caterpillars. The Hotelling’s T2 ellipse confines the confidence region (95%) of the score plot. d) The loading plot defining the contribution of each of the volatile compound to the orientation of sample groups in the score plot. For compound identity see Table 1. Abbreviations used: Cg-ST = starved parasitised caterpillar; Cg-ST+EMD = starved then external microbiome disrupted parasitised caterpillar. (TIF) [file ppat.1011262.s003.tif]

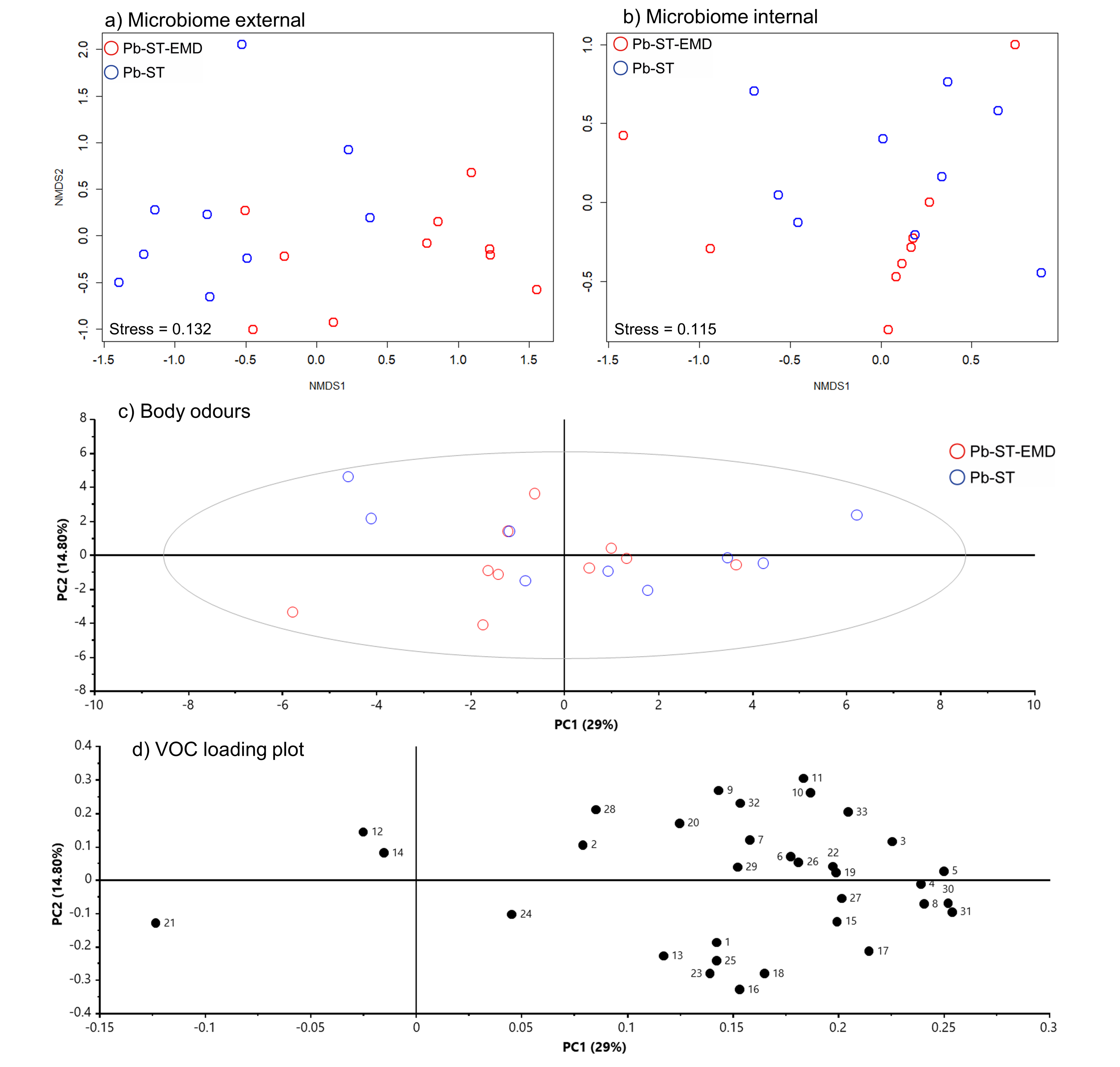

Supplement: S4 Fig — a) NMDS ordination plots based on Bray–Curtis distances of Hellinger-transformed relative abundance data of the external (dermal) bacterial communities (stress = 0.132). b) NMDS ordination for the internal bacterial communites (stress = 0.115). c) PCA plot for the volatile blends of different groups of caterpillars. The Hotelling’s T2 ellipse confines the confidence region (95%) of the score plot. d) The loading plot defining the contribution of each of the volatile compound to the first two principal components. For compound identity see Table 1. Abbreviations used: Pb-ST = starved unparasitised caterpillar; Pb-ST+EMD = starved then external microbiome disrupted unparasitised caterpillar. (TIF) [file ppat.1011262.s004.tif]

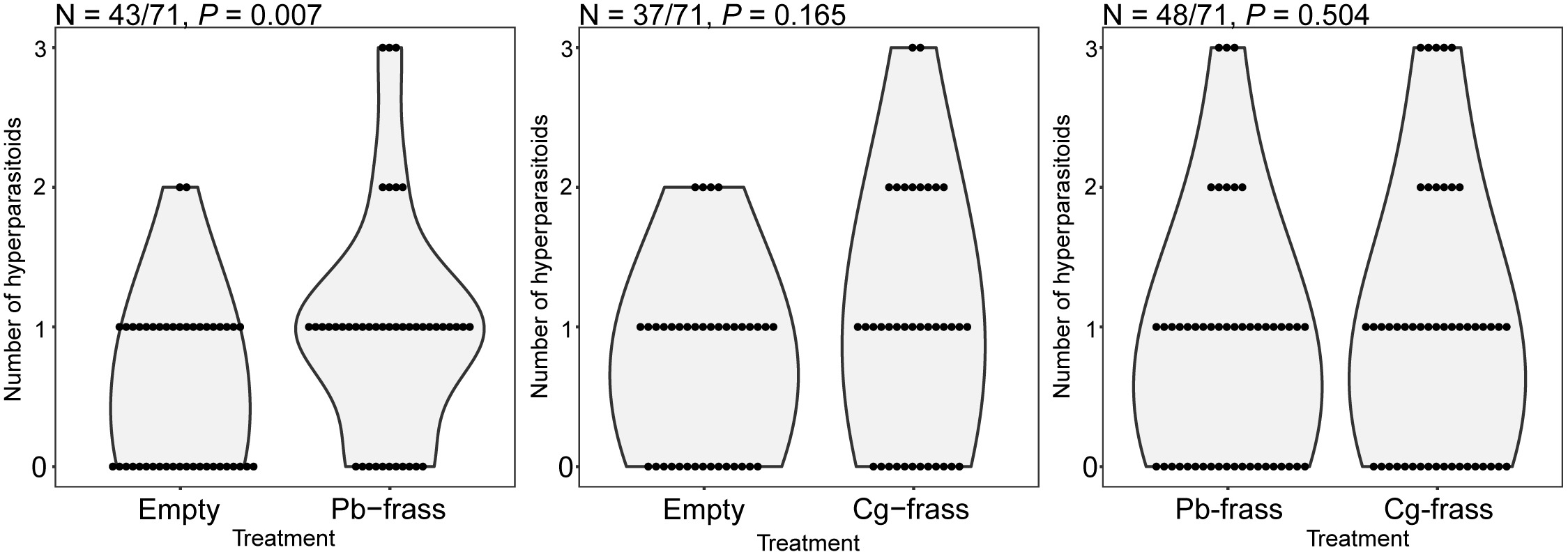

Supplement: S5 Fig — The number of B. galactopus found inside a chamber of the two-chamber olfactometer after one hour (out of 5 individuals). Chambers were either empty or contained fresh frass from L5 unparasitised or parasitised caterpillars. N = the number of replicates, for each replicate 5 B. galactopus were released in the olfactometer. P was calculated using a Mann-Whitney U-test. (TIF) [file ppat.1011262.s005.tif]

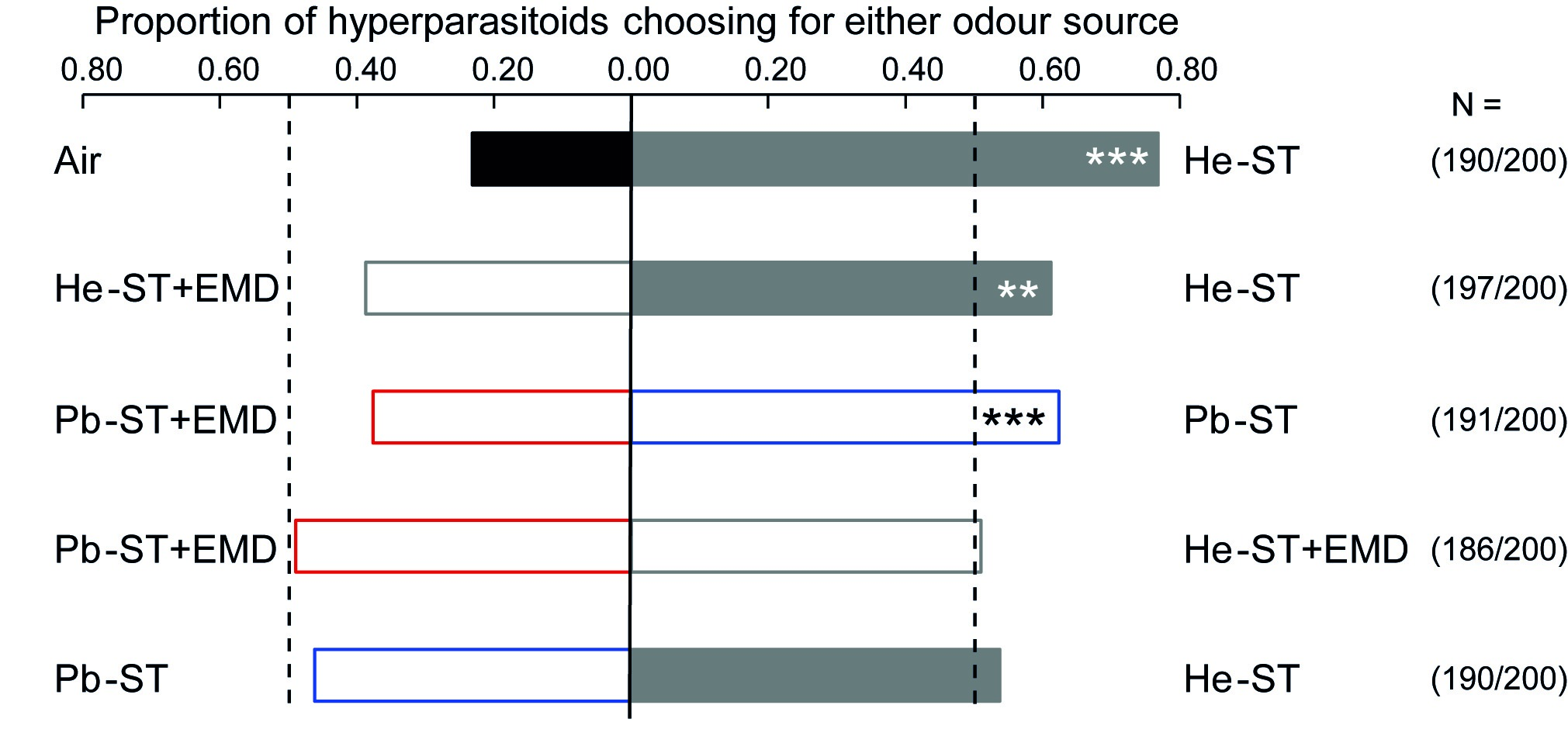

Supplement: S6 Fig — Preference of the hyperparasitoid Baryscapus galactopus for caterpillar body odours was tested with Y-tube olfactometer tests. Tested combinations were selected according to the hypothesis that hyperparasitoids can use caterpillar body odours, which are (at least partially) determined by the external microbiome. Numbers between brackets indicate the number of wasps that made a choice within 10 min from the start of the experiment versus the total number of wasps tested. *** P <0.001; two-sided binomial test. Abbreviations used: AIR = clean air; HE = Untreated parasitised caterpillar; HE-ST = starved parasitised caterpillar; HE-ST+EMD = starved then external microbiome disrupted parasitised caterpillar. Pb = Untreated unparasitised caterpillar; Pb-ST = starved unparasitised caterpillar; Pb-ST+EMD = starved and external microbiome disrupted unparasitised caterpillar. (TIF) [file ppat.1011262.s006.tif]

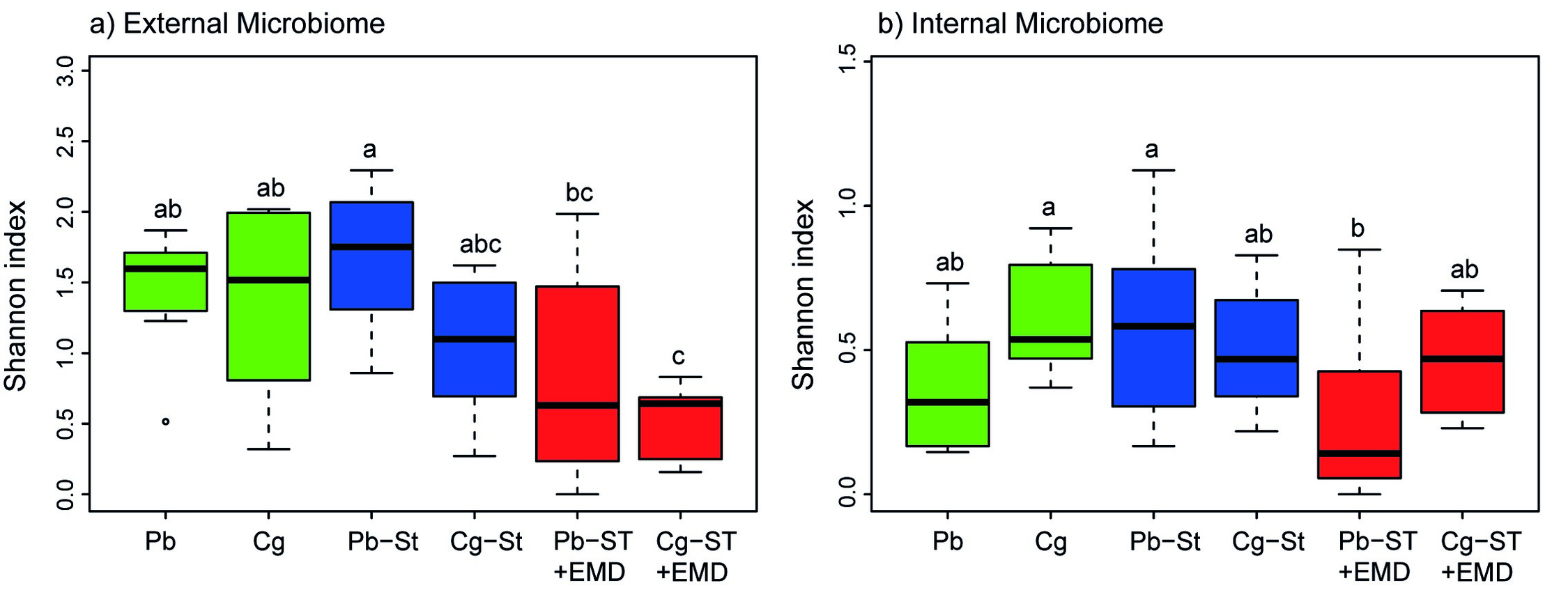

Supplement: S7 Fig — a) The shannon diversity of the external microbiome b) The Shannon diversity of the internal microbiome. (TIF) [file ppat.1011262.s007.tif]

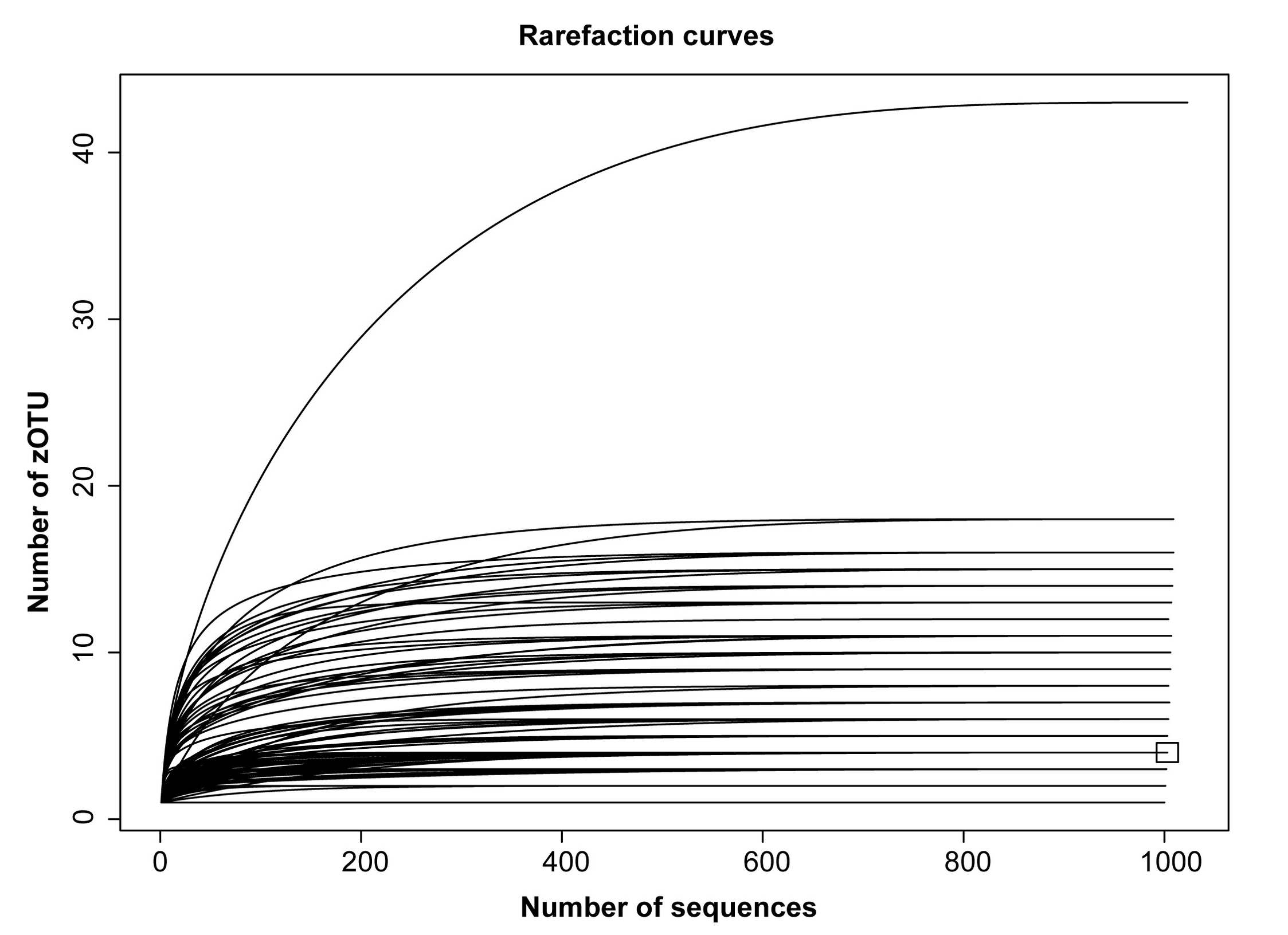

Supplement: S8 Fig — Rarefaction curves approached saturation, indicating that our sequencing depth was sufficient to cover the microbial diversity. (TIF) [file ppat.1011262.s008.tif]
