# Supplementary material for: Parasitism causes changes in caterpillar odours and associated bacterial communities with consequences for host-location by a hyperparasitoid
Source: PLoS Pathog. 2023 Mar 22;19(3):e1011262. doi: 10.1371/journal.ppat.1011262 (PMC10069771; doi:10.1371/journal.ppat.1011262)
Supplement: Attachment — Submitted filename: 20230218PPat_Rebuttal_FINAL.docx [file ppat.1011262.s010.docx]

Dear Bourne,

Thank you very much for submitting your manuscript "Parasitism causes changes in caterpillar odours and associated bacterial communities with consequences for host-location by a hyperparasitoid" for consideration at PLOS Pathogens. As with all papers reviewed by the journal, your manuscript was reviewed by members of the editorial board and by several independent reviewers. In light of the reviews (below this email), we would like to invite the resubmission of a significantly-revised version that takes into account the reviewers' comments.

Based upon my own reading of the manuscript and the consensus of the three reviewers, this is a very interesting and well-written study of the odors involved in parasitism by hyperparasitoids. The attempts of the authors to investigate the origin of odors that are attractive to hyperparasitoids was appreciated. The research topic is fascinating. I agree with the reviewers that some substantial revisions are necessary. In the aggregate, the three reviewers outline several major issues that the authors will need to address, and I agree with these. I consider that the most crucial of these are:

**A:** Dear editor, thank you for your kind words and your enthusiasm for our work and for your summary of key points. We have carefully considered the reviewers comments and incorporated them to the best of our ability.

1.) The surface sterilization procedure appears to be inefficient or not effective, leading to increases of some taxa. Reviewer 1 suggests measuring volatiles in parasitized caterpillars before and after surface-sterilization.

**A:** We acknowledge that the sterilisation procedure was not 100% effective. The dosage used for sterilisation was already testing the limits considering caterpillar mortality and/or effects on hyperparasitoid behaviour. Higher dosages of sodium hypochlorite could not be used. We agree that our method thus did not achieve surface sterilisation and we altered surface sterilisation throughout the text to “External microbiome disruption”. (“to modify the absolute and relative abundance of some microorganisms” sensu reviewer 3). Our data show that the microbiome after the hypochlorite treatment was substantially altered and affected hyperparasitoid behaviour. In the revised manuscript we clarify the treatment in more detail.

Furthermore, we agree with the reviewer that a measurement of volatiles in (untreated) parasitized caterpillars before and after external microbiome disruption would be of added value. We intended to include this treatment in the original design of the study. This was ultimately excluded because un-starved caterpillars were shown to defecate and regurgitate excessively during the washing treatment in pilot studies. The volatile blend of these caterpillars would thereby be largely characterised by faeces and compounds associated with regurgitate that have a strong smell.

2.) The fact that the hyperparasitoid assay shows that the hyperparasitoid does not discriminate between parasitized and unparasitized caterpillars is problematic given the title of the paper and sections of the discussion. Perhaps this can be addressed and or clarified, but it does seem to diminish the significance of the present study, given that the authors have already published the effects of parasitism on the microbiome of *P. brassicae* (Gloder et al., 2021)

**A:** We added new data to support our hypothesis regarding the behaviour of the hyperparasitoid and nuance our conclusions based on our findings in the opening of the discussion (Line 343-343). We provide direct evidence of a role for *Wolbachia* in the response of hyperparasitoids to parasitized caterpillars.

3.) It is pointed out that several conclusions are not currently supported by the data.

**A:** In our revised manuscript we more carefully phrased the conclusions on hyperparasitoid behaviour to not overreach our data. The first section of the discussion has now been fully rewritten to reflect the results better:

**L343-346:** In this study we show that parasitism by *C. glomerata* leads to a significant alteration of the odours of its host caterpillar *P. brassicae,* in which five VOCs are considered to be induced after parasitism. This aligns with changes in the internal and external bacterial communities, hinting at a relationship between caterpillars’ body odours and their microbiome. Moreover, hyperparasitoids preferred the odours of starved, parasitised caterpillars (Cg-ST) over odours from starved then external microbiome disrupted parasitised caterpillars (Cg-ST+EMD), indicating a role of bacteria and/or their volatiles in the host-location of hyperparasitoids.

4.) The hypothesis that *Wolbachia* sp. Is the driver of mVOC changes in parasitized caterpillars should be explored in more detail.

**A:** We took the challenge to provide direct evidence for the role of *Wolbachia* in the interaction network. We explored the behaviour of the hyperparasitoid studied after injecting *Wolbachia* in the caterpillar. In particular, we observed that the injection of *Wolbachia* in unparasitized caterpillars enhanced hyperparasitoid attraction (time to make first contact) comparable to the response seen when presenting them a parasitized caterpillar. These important results have been incorporated in the manuscript and strengthen the conclusions and value of our study (Lines 401-433).

### Part I – Summary

Please use this section to discuss strengths/weaknesses of study, novelty/significance, general execution and scholarship.

Reviewer #1: Bourne et al investigated how parasitic wasp affect the emitted volatiles and the microbiome of their host, and how these changes impact the foraging behavior of hyperparasitoids. Through the characterization of insect microbiomes, volatile measurements, and behavioral experiments, and different statistical analyses, the authors conclude that that parasitism by *C. glomerata* leads to a significant alteration of the odours of its host caterpillar *P. brassicae* and that the observed odour changes are related to changes in the internal and external microbiome of the caterpillars, and in turn determine hyperparasitoid preferences.

I find that the research topic is fascinating, but have major concerns as described below:

1. The authors conclude (Line 343) that: “In this study we show that parasitism by *C. glomerata* leads to a significant alteration of the odours of its host caterpillar *P. brassicae*. This statement is not supported by the data. Only three volatiles, (Z)-4-Hepten-1-ol, 3-Octanol, and 3-Octanone are significantly, and consistently induced upon parasitization. However, several compounds seem to be suppressed, but there is no statistical support for this statement nor these changes are always consistent across treatments.

**A:** Thank you for you kind words and constructive criticism on our work. Indeed, only few volatile compounds are altered significantly when comparing individual compounds between all treatments, but the large difference between volatile blends is poorly reflected by this. This is shown in the PERMANOVA analysis (Line 160-161; 164-165), which points at statically significant differences between treatments (*F*_2,53_ = 17.292*, p <* 0.001) and parasitism statuses (*F*_1,53_ = 4.594*, p* = 0.011). Thus, the multivariate analysis of the volatile blend provides the evidence, whereas only few individual compounds on their own were significantly altered. This suggests that not individual compounds but changes in blends are used by hyperparasitoids in host location (similar to what is known for many primary parasitoids). Hence, we still stand behind our statement in Line 343: “Parasitism by *C. glomerata* leads to a significant alteration of the odours of its host caterpillar *P. brassicae*”.

However, based on your and other reviewers’ comments we now clarify our usage of PERMANOVA in the comparison of volatile blends in the results section (Line 160-161; 164-165), mention in the results section under which conditions we consider a VOC to be induced upon parasitisation (Line 163) and emphasize that only few (with our newly formulated conditions five) individual compounds are induced upon parasitisation in the first paragraph of the discussion (Line 343):

**Line 343:** ..., in which five VOCs are considered to be induced after parasitism.

**Line 163:** A compound was considered characteristic for parasitised caterpillars when it was significantly increased for parasitised caterpillars in at least one pairwise comparison with unparasitised caterpillars undergoing the same treatment (e.g. Cg-ST and Pb-ST).

**Line 161:** .. (PERMANOVA: *F*_2,53_ = 17.292*, p <* 0.001)

**Line 165:** .. (PERMANOVA: *F*_1,53_ = 4.594*, p* = 0.011)

**Line 161:** ... top four VOCs in VIP-score (3-octanone, (Z)-3-hepten-1-ol, 3-Methyl-3-buten-1-ol and 3-octanol) and ...

**Line 167-168: ...** highlight the importance of 3-octanone, (Z)-34-hepten-1-ol, 3-Methyl-3-buten-1-ol, 3-octanol and 3-pentanone in characterising parasitism status of caterpillars.

**Line 358-359:** The VOCs (Z)-3-hepten -1-ol, 3-octanol, 3-pentanone, 3-Methyl-3-buten-1-ol and 3-octanone were characteristic ...

**Line 363-364:** 3-Methyl-3-buten-1-ol an olfactory-active compound to a coccinellid predator to locate and perceive its aphid prey [48].

**Line 383-386:** Four out of four five characteristic VOCs for parasitised caterpillars are known to be mVOCs according to the mVOC 3.0 database [50]. 3-Pentanone, 3-methyl-3-buten-1-ol and 3-octanone are produced by a variety of bacteria and fungi ...

Furthermore, we adjusted throughout the manuscript that conditions five individual compounds are induced upon parasitisation instead of four.

2. The authors conclude (Line 344) that: The odour changes corresponded with changes in the internal and external microbiome of the caterpillars. This statement is not supported by the data. To fully support this statement, authors should measure volatiles in parasitized caterpillars before and after surface-sterilization. The authors conducted some of these measurements, although with starved larvae, which limits the interpretation of the results.

**A:** We agree with the reviewer that this statement should ideally be supported by a measurement of volatiles in (untreated) parasitized caterpillars before and after surface-sterilization. We intended to include this treatment in the original design of the study, but this it was excluded because un-starved caterpillars were shown to defecate and regurgitate excessively during the washing treatment in pilot studies, reducing the effectiveness of the surface sterilisation (renamed to “external microbiome disruption”) treatment.

We now clarify in the method section that only starved caterpillars were subjected to the washing treatment to limit contamination with bodily products (Line 523). Furthermore, we nuanced our statement on whether odour changes correspond with changes in the internal and external microbiome of caterpillars (Line 344):

**Line 344:** This aligns with changes in the internal and external bacterial communities, hinting at a relationship between caterpillar body odours and microbiome.

**Line 523:** Only starved caterpillars were subjected to the washing treatment to limit excessive contaminations with bodily products (frass and regurgitate).

Moreover, the surface sterilization procedure seems to be inefficient as there are some bacteria that increase (zOTU1 and zOTU2, some of the most abundant ones) after surface sterilization (Fig. 3). Apart from this, there is again no statistical support for this statement.

**A:** We agree with the reviewer that the surface sterilisation treatment is inefficient considering that this treatment does not achieve full sterility as the name suggests. However, in the light of our study we opted to use a “sterilisation” treatment as a crude measure to disrupt the external microbiome of (parasitised) caterpillars to study whether mVOCs are altered. In our biological context, sterility is hard to achieve within the organismal limits of caterpillars and would lead to undesired mortality and thus the presence of decomposition-related odours. To remove the expectations and implications surrounding the surface sterilisation treatment, we renamed this treatment to “External microbiome disruption” (with the abbreviation EMD) throughout the manuscript. Furthermore, we clarify the exact purpose of the “External microbiome disruption” method at the start of the results (L149-150) and materials and methods sections (L552):

**Line 149-150:** … and starvation followed by disruption of the external microbiome (referred to as external microbiome disruption treatment) to additionally modify the absolute and relative abundance of some microorganisms in the external microbiome of caterpillars

**Line 522:** This disruption reduces the absolute and relative abundance of most external microorganisms as a consequence of the different sensitivity to sodium hypochlorite and the differences in the adhesive capacity of bacteria [69,70]

There are significant changes across treatments, especially starvation has a drastic effect on volatile emissions, but volatile levels emitted by control and surface sterilized insects are not statistically different regardless of their starvation or parasitization status (Table 1). What the authors can say about this volatile data is that starvation drastically impact volatile emissions by the caterpillars.

**A:** We agree with your observation that generally, the emissions of single compounds do not differ much between unparasitised and parasitised caterpillars of the same treatment (e.g. Cg-ST and Pb-ST). And that the strongest effects are due to starvation (and thus frass-related; new figure 2b). However, the selected compounds related to parasitism ((Z)-3-Heptenol, 3-Octanol, 3-Pentanone, 3-Methyl-3-buten-1-ol and 3-Octanone) all do have at least one significant difference within the same treatment which indicates that they are related to parasitism. We further clarify that the trends are consistent, and not necessarily all pairwise comparisons are significantly different from one other (L347). Finally, we elaborate under which conditions volatile compounds are considered to be related to parasitism (Line 163). Due to the defined criteria, also 3-Methyl-3-buten-1-ol is considered to parasitism-related compound. This is adjusted throughout the manuscript:

**Line 347:** ... identified some consistent patterns in alterations of parasitised caterpillar odours regardless of whether caterpillars were starved (ST) or starved then external microbiome disrupted (ST+EMD)

**Line 163:** A compound was considered characteristic for parasitised caterpillars when it was significantly increased for parasitised caterpillars in at least one pairwise comparison with unparasitised caterpillars undergoing the same treatment (e.g. Cg-ST and Pb-ST).

**Line 161:** ... top four VOCs in VIP-score (3-octanone, (Z)-3-hepten-1-ol, 3-Methyl-3-buten-1-ol and 3-octanol) and ...

**Line 167-168: ...** highlight the importance of 3-octanone, (Z)-3-hepten-1-ol, 3-Methyl-3-buten-1-ol, 3-octanol and 3-pentanone in characterising parasitism status of caterpillars

**Line 358-359:** The VOCs (Z)-3-hepten -1-ol, 3-octanol, 3-pentanone, 3-Methyl-3-buten-1-ol and 3-octanone were characteristic ...

**Line 363-364:** 3-Methyl-3-buten-1-ol an olfactory-active compound to a Coccinellid predator to locate and perceive its aphid prey [48].

**Line 383-386:** Four out of four five characteristic VOCs for parasitised caterpillars are known to be mVOCs according to the mVOC 3.0 database [50]. 3-Pentanone, 3-methyl-3-buten-1-ol and 3-octanone are produced by a variety of bacteria and fungi ...

3. Lastly, the authors conclude (Line 345) that the odour changes corresponded with …attraction of hyperparasitoids to parasitized caterpillars. Again, this statement is not supported by the data. Hyperparasitoids do not show any preference for parasitized over unparasitised caterpillars independently of surface sterilization treatments (See the results of the choice experiments between Pb-ST+W vs Cg-ST+W, and also between Pb-ST vs Cg-ST). What the authors can show is that hyperparasitoids preferred the odours of starved, parasitised larvae (Cg-ST) over odours from surface sterilized caterpillars (Cg-ST+W)(Fig 5), pointing to a potential role of bacteria (or their volatiles).

**A:** Thank you for your comment and suggestion; we agree that our wording is too optimistic and have reworded this sentence in line 345 in accordance with your suggestion to better reflect the data shown:

**Line 345:** Moreover, hyperparasitoids preferred the odours of starved, parasitised caterpillars (Cg-ST) over odours from starved then external microbiome disrupted parasitised caterpillars (Cg-ST+EMD), indicating a role of bacteria and/or their volatiles in the host-location of hyperparasitoids.

Taken together, there seems to be additional factors (independently of microbial volatiles) that differ between surface sterilized, starved, parasitized caterpillars (Cg-ST+W) and non-sterilized, starved, parasitized caterpillars (Cg-ST) that can explain the preference of hyperparasitoids.

**A:** Based on these suggestions, we took the challenge to provide direct evidence of a role for *Wolbachia* in the interaction. After injection of *Wolbachia* into caterpillars of *Pieris*, we found that hyperparasitoid behaviour was significantly altered. Hyperparasitoids made faster first contact with *Wolbachia*-injected caterpillars compared to untreated control caterpillars, while no differences were found compared to parasitized caterpillars, suggesting that *Wolbachia* has a significant role in hyperparasitoid host location. However, the total mounting time did not differ from the control treatment, and was less than the time spent on parasitized caterpillars. These results have now been added to the manuscript, see Materials and Methods, Lines 900-947 and Results, Lines 401-433. Below the corresponding figure is presented.

**Fig 7. Results of the no-choice assay to assess the role of *Wolbachia* in hyperparasitoid host location.** Bar plots showing (a) the average time (±SE) until first contact of *B. galactopus* with the caterpillar host *P. brassicae*, and (b) total mounting time (±SE) of *B. galactopus* on the caterpillars within 1 h after first contact was made. Hyperparasitoids were subjected to four treatments, including unparasitised caterpillars (Unparasitised-PBS; *n* = 39), caterpillars parasitised by *C. glomerata* (Parasitised-PBS; *n* = 36), caterpillars injected once with *Wolbachia* (Wolb-PBS; *n* = 50), and caterpillars injected twice with *Wolbachia* (Wolb-Wolb; *n* = 46). Statistical differences among treatments are indicated with different letters based on Kruskal-Wallis tests with Dunn’s test for multiple comparisons including a Hochberg correction. Pie charts show the percentage of responding (black) and non-responding (grey) hyperparasitoids.

Minor comments:

1. Table 1. It is very difficult to actually see what are the volatiles that differ across treatments. I suggest that this table is moved to supplementary material and that the authors create bar plots for the volatiles that are statistically different across treatments, specially those that are relevant to interpret the results of the choice experiments.

**A:** Thanks for your suggestion to increase the clarity, we added jittered bar plots for the relevant compounds as suggested (Fig. 2abc). Furthermore, we made some textual edits to refer to this new Figure 2. Consequently the numbering of all figures changed in the revised manuscript (n + 1 except Figure 1).

**Line 185 (graph on next page):**

**Fig 2. Volatile compounds (tentative identification) detected in the headspace of untreated, starved or starved then external microbiome disrupted unparasitised or *C. glomerata* parasitised caterpillars (*P. brassicae*) with a Variable Importance in the Projection (VIP) values > 1 for the OPLS-DA.** Amounts of individual compounds are given as the average of peak height /104 (±SE). are given. Statistical differences among treatments for compounds with VIP score > 1 are indicated with different letters based on Kruskal-Wallis tests with Dunn’s test for multiple comparisons including a Bonferroni correction. a) Compounds in yellow background are correlated with parasitism status. b) Compounds in green background are correlated with the presence/absence of frass. c) Compound in blue background are not correlated to either of these. Abbreviations used: Pb = Untreated unparasitised caterpillars; Cg = Untreated parasitised caterpillars; Pb-ST = starved unparasitised caterpillars; Cg-ST = starved parasitised caterpillars; Pb-ST+EMD = starved and external microbiome disrupted unparasitised caterpillars. Cg-ST+EMD = starved and external microbiome disrupted parasitised caterpillars.

2. The last paragraph of the introduction should be shorten and should be focused more on a short summary of the hypothesis being tested, a very brief summary of the main findings and put them in a broader context. Right now, the authors describe too many detailed results.

**A:** We shortened the final paragraph of the introduction to remove the surplus of details on the results, while including the results of the injection experiment. Furthermore we added a new final sentence to place our findings in a broader context (L133).

**Removed Line 123**: ... aiming to remove transient internal (frass-related) and skin mVOCs, ...

**Removed Line 125-127:** This suggests a role of the resident internal microbiome, which remained stable after starvation and external microbiome disruption, in determining the caterpillar odour profile.

**Removed Line 129:** .. after parasitism that were most abundant in the internal microbiome.

**Line 130 added**: Furthermore, we found that the hyperparasitoid B. galactopus preferred the odours of parasitised caterpillars with an intact external microbiome over caterpillars with a disrupted external microbiome. Moreover, injection of *Wolbachia* sp. into unparasitised caterpillars resulted in enhanced attraction of hyperparasitoids, shown by a shorter time to first contact with the *Wolbachia*-injected caterpillars compared to mock-injected caterpillars.

**Line 133 added:** These results indicate that parasitoid wasps reveal themselves indirectly to their hyperparasitoid natural enemies by modifying the bacterial communities of their host.

3. In the summary. I would write “The mVOCs might thereby provide a reliable cue to carnivorous enemies in locating their host or prey. “Parasitism by parasitoid wasps might alter the microbiome of their caterpillar host, affecting organismal odours and interactions with insects of higher trophic levels”.

**A:** Thank you for this suggestion, we applied this edit which adds nuance to the abstract in an elegant way.

**L18:** the mVOCs might thereby provide …

**L20:** … parasitoid wasps might alter the microbiome …

Reviewer #2: In this manuscript, ‘Parasitism causes changes in caterpillar odours and associated bacterial communities with consequences for host-location by a hyperparasitoid’, the authors present a combination of chemical, microbial, and behavioral experiments to address the question of whether parasitism of Pieris brassicae caterpillars by the endoparasitoid *Cotesia glomerata* (or Hyposoter ebeninus) affects the microbial community, odor, and attractiveness of caterpillars to the hyperparasitoid Baryscapus galactopus. The authors describe previous work in a related caterpillar (*Pieris rapae*) that detected changes in caterpillar body odor that allow hyperparasitoids to located parasitized caterpillars, and work in Pieris brassicae (the focal species in this study) demonstrating changes in the internal microbiome after being parasitized. This manuscript aims to address whether odors that attract hyperparasitoids are produced by the caterpillar, caterpillar frass, or are microbial in origin (internal/external microbiome). This is a very interesting manuscript that represents a significant effort (particularly the sample sizes for the y-tube assay), however I did note several areas of concern.

**A:** Thanks for your constructive criticism on our work. You accurately summarized our starting point and goals.

Reviewer #3:This paper deals with the role of microorganisms as drivers of interaction between insects at multiple trophic levels. Specifically, evidence is provided that parasitism of the large cabbage white caterpillar Pieris brassicae by endoparasitoid wasp *Cotesia glomerata* led to the production of four characteristic volatile products and significantly affected the internal and external microbiome of the caterpillar, which were both found to have a significant correlation with caterpillar odors, with consequences for host-location by the hyperparasitoid Baryscapus galactopus. It was hypothesized that the changes in external microbiome and body odour after parasitism were driven by the resident internal microbiome of caterpillars, where the bacterium *Wolbachia* sp. was only present after parasitism.

The paper is well written and analyzes the role of the microbiota in a very interesting biological host-parasite interaction system. The study, however, appears to be mainly observational and also contains some incongruences. This unfortunately diminishes the enthusiasm in reading it. In particular, the hypothesized involvement of *Wolbachia* as a main driver in the system (as it results from forward selection redundancy analyses), is attractive, but deserves further investigation from a mechanistic point of view to strengthen the paper. Therefore, while containing elements of originality and novelty, the study appears, in my opinion, preliminary in its current state.

**A:** Thank you for the kind words and constructive criticism on our work. We agree that our work was mostly providing corresponding/correlative evidence and utilised the comments of all three reviewers and the editor to remove the incongruences. In addition, we explored the role of *Wolbachia* more intensively to add more depth to our study and strengthen our conclusions with causal evidence for the role of *Wolbachia*. As mentioned above, results showed that micro-injection of healthy caterpillars with *Wolbachia* increased hyperparasitoid attraction (time to make first contact) to a level similar with attraction to parasitized caterpillars, suggestion an important role of *Wolbachia* in hyperparasitoid behaviour. These results have been added in Lines 401-433 while the experimental set-up is described in Lines 900-947.

**Part II – Major Issues: Key Experiments Required for Acceptance**

Please use this section to detail the key new experiments or modifications of existing experiments that should be absolutely required to validate study conclusions. Generally, there should be no more than 3 such required experiments or major modifications for a "Major Revision" recommendation. If more than 3 experiments are necessary to validate the study conclusions, then you are encouraged to recommend "Reject".

Reviewer #1: (No Response)

Reviewer #2:There are two major concerns related to the results presented in this manuscript:

1) Statistical analysis of volatile data – The authors state (lines 582 through 585), “Supervised orthogonal partial least squares-discriminant analysis (OPLS-DA) and unsupervised principal component analysis (PCA) statistical models were used as a tool to compare and correlate treatment groups. Whereas PCA was used when no significant model for OPLS-DA could be obtained.

”For OPLS-DA the methods state that R2 and Q2 metrics were calculated and models were validated using permutations and CV-ANOVA. I do not see the model metrics and validation results reported in the manuscript. The variable importance scores (VIP) from OPLS-DA are the basis for many conclusions in this manuscript, but currently there is no information presented about the validity of the models.

Also related to analysis of volatile data, the results report p values from comparisons between treatments (e.g. line 161, line 165). Neither OPLS-DA nor PCA is a statistical test that produces p-values, and the methods do not appear to list other analyses that were conducted on the volatile data. It is not clear to me what test generated these results. Please clarify this, and any areas where p-values or other metrics are reported without indicating origin.

**A:** Thanks for your detailed comments and suggestions to improve the text, we agree that R2 and Q2 metrics were missing and added these to the results (Line 163) and applicable supplementary graphs (S1-2 Fig). Furthermore, we clarify that the p-values of comparisons between treatments originate from PERMANOVA analyses (Line 161, 165) and clarified the origin of p-values and other metrics when missing in other sections. For pairwise comparisons between treatments for single VOCs we used Dunn’s test, as mentioned in the table caption. We adjusted the manuscript to clarify this in the material and methods as well (Line 593). Re-running this analysis in R, resulted in slightly different results compared to the previous table. We updated the table accordingly, in general the conclusions remain the same.

**Line 156:** This dataset was subjected to orthogonal partial least squares discriminant analysis (OPLS-DA), which yielded a significant model (CV-ANOVA: p < 0.001, R2 = 0.344 ; Q2 =0.276), with two predicting components accounting for 33.70%, 6.81% and 20.50% of the total of the total variance, respectively.

**Line 161:** .. (PERMANOVA: *F*_2,53_ = 17.292*, p <* 0.001)

**Line 165:** .. (PERMANOVA: *F*_1,53_ = 4.594*, p* = 0.011)

**Line 226:** ... the internal (PERMANOVA, F_1,53_ = 4.865, *p* = 0.014)

**Line 234:** ... the external bacterial community (PERMANOVA, F_2,53_ = 5.716, *p* < 0.001)

**Line 238:** ... affected by treatment (PERMANOVA, F_2,53_ = 1.083, *p* = 0.341)

**Line 248:** affected by parasitism (external: F_1,53_ = 5.584, P = 0.022 ; internal: F_1,53_ = 4.379, *p* = 0.041; ANOVA)

**Line 251:** internal microbiome (external: F_2,53_ = 12.661, P < 0.001 ; internal: F_2,53_ = 2.394, *p* = 0.062; ANOVA)

**Line 276-277:** external (ANOVA-like permutation test: F_5,33_ = 2.704, R² = 0.29, *p* = 0.001) and internal bacterial communities (ANOVA-like permutation test: F_4,34_ = 2.99, R² = 0.26, *p* = 0.001)

**Line 593:** After identifying compounds with a variable importance in the projection (VIP) score of 1 or higher as potentially relevant, we subjected these to further analyses using a Kruskal-Wallis test followed by Dunn's test for multiple comparisons with a Bonferroni correction to determine significant differences between treatments. This was done using the dunn.test command in R-studio (dunn.test package) [73,74].

2) The bioassay results indicate that hyperparasitoid females did not exhibit any preference for parasitized caterpillars when given the choice between parasitized and unparasitized caterpillars. These bioassays were conducted a second time using another parasite species and the results were consistent. The outcome of these bioassays is summarized in lines 329-331, “The consistency of these results indicate that the preference of the hyperparasitoid is corelated with the presence of the external microbiome, though it is not discriminating between the odours of parasitised and unparasitised caterpillars in this setup.

”However, the discussion opening (line 343-346) states that, “…*C. glomerata* leads to a significant alteration of the odours of its host caterpillar *P. brassicae*. The odour changes corresponded with changes in the internal and external microbiome of the caterpillars and attraction of hyperparasitoids to parasitized caterpillars.

”Given these results, I do not understand logic behind this statement in the discussion. The hyperparasitoids appear to prefer an intact external microbiome regardless of of parisitism status, and direct comparisons testing the effect of parasitism status (Pb-ST vs. Cg-St and Pb-ST-W vs. Cg-ST-W) were non-significant.

**A:** We agree with you, the other reviewers and the editor that the discussion opening was written too optimistic in the previous version and we revised the first paragraph to not overreach our data:

**L343-346:** In this study we show that parasitism by *C. glomerata* leads to a significant alteration of the odours of its host caterpillar *P. brassicae*, in which five VOCs are considered to be induced after parasitism. This aligns with changes in the internal and external bacterial communities, hinting at a relationship between caterpillars’ body odours and their microbiome. Moreover, hyperparasitoids preferred the odours of starved, parasitised caterpillars (Cg-ST) over odours from starved then external microbiome disrupted parasitised caterpillars (Cg-ST+EMD), indicating a role of bacteria and/or their volatiles in the host-location of hyperparasitoids. The role of the internal microbiome was confirmed by the enhanced attraction of the hyperparasitoid to unparasitised caterpillars injected with *Wolbachia* over mock-injected caterpillars.

This confusion also occurs when reading lines 428-430 versus lines 440-441. Line 428-430: “…our results support the hypothesis that the changes after parasitism in the microbiome and caterpillar odour provide a mechanism by which hyperparsitoids can locate or distinguish hosts.

”Line 440-441: “It is remarkable that the hyperparasitoid B. galactopus is unable to discriminate between starved parasitised and unparasitised *P. brassicae* caterpillars based on their odours.

”Without more detailed explanation, the discussion appears contradictory. Please clarify these areas and provide more details related to the interpretation of results.

**A:** We clarified the suggested sections to remove the possible confusion and provide more details in interpretation of the results. Moreover, an additional experiment has been performed and added to the manuscript to further show how *Wolbachia* affects hyperparasitoid behaviour (see also above). Results of this additional experiment have been added in Lines 401-433, while the experimental set-up is described in Lines 900-947 and results are discussed in Lines 531-553.

**Removed line 443:** This is despite the finding that *P. brassicae* emits a more pronounced parasitism signal in volatiles than *P. rapae*.

**Added Line 443:** The hyperparasitoid *B. galactopus* was unable to discriminate between starved parasitised and starved unparasitised *P. brassicae* caterpillars based on their odours, as opposed to previous findings in *P. rapae*. Possibly, the starvation treatment applied in our current study with *P. brassicae* reduced the differences between parasitised and unparasitised caterpillars on which hyperparasitoids rely. These manipulations were not performed in our previous study on *P. rapae* [38]. The compound 2,3-butadione could play an important role in the ability of hyperparasitoids to discriminate between healthy and parasitised caterpillars as shown for *P. rapae* [38].

Reviewer #3:

1. Microbiome manipulation treatment. "Surface sterilization" (Ln. 520-523) has not been microbiologically verified. Gentle treatment of caterpillars (three washes with 0.05% sodium hypochlorite) is unlikely to be sufficient to "sterilize". Such a treatment could be sufficient to modify the relative abundance of some microorganisms as a consequence of the different sensitivity to sodium hypochlorite and adhesive capacity.

**A:** Your observation is correct in the sense that our surface sterilisation treatment did not lead to bacteria-free caterpillars. We were limited by the biology of parasitised caterpillars as more effective sterilisation techniques lead to caterpillar mortality. Throughout the manuscript we changed “surface sterilisation” to “External microbiome disruption” (with the abbreviation EMD). Furthermore, we clarify the exact purpose of the “External microbiome disruption” method at the start of the results (L149-150) and materials and methods sections (L552).

**Line 149-150:** ... and starvation followed by disruption of the external microbiome (referred to as external microbiome disruption treatment) to additionally modify the absolute and relative abundance of some microorganisms in the external microbiome of caterpillars

**Line 522:** This disruption reduces the absolute and relative abundance of most external microorganisms as a consequence of the different sensitivity to sodium hypochlorite and the differences in the adhesive capacity of bacteria [69,70].

Thus, the “decline of Staphylococcus sp. (zOTU 3) and increase of Enterococcus sp. (zOTU 1) relative abundances” (ln. 236) could be simply due to this effect. As a consequence, the suggestion that “the internal microbiome potentially acted as a source for the external microbiome” (242-243) is probable but not supported, also because Enterococcus sp. (zOTU 1) and Enterobacteriaceae sp. (zOTU 2) appear to be present and abundant in the external bacterial community even without surface sterilization treatment (Fig. 3d).

A: We agree that the statement that “the internal microbiome potentially acted as a source for the external microbiome” is too suggestive and serves no further purpose in the manuscript. Thus, we removed line 240-243.

2. The result of forward selection redundancy analyses (RDA) (Fig. 4) is rather weak. A robust correlation between microorganisms and odors appears to be missing, with the exception of *Wolbachia*. However, this correlation is obvious, given that *Wolbachia* is transferred upon oviposition from the parasitoid into the caterpillar, and therefore its presence is intrinsically associated with *C. glomerata* parasitism. Probably the only relevant result is the reduction of Sphingomonas in the parasitized caterpillars, which should be better explored in this study.

**A:** We agree that besides the obvious correlation with *Wolbachia*, the reduction of *Sphingomonas* in parasitised caterpillars is the most interesting finding of the RDA analysis. Hence, we already explore this finding in L402-408 of the manuscript. We now clarify that our amplicon sequencing does not allow us to explore the genome of this bacterium more in depth and further elaboration on the role of *Sphingomonas* in the production of mVOCs would require fermentations with this bacterium. However, fermentations with individual bacteria are beyond the scope of this study and were thus not performed.

**Line 408:** The performed amplicon sequencing does not allow us to explore the genome of the *Sphingomonas* bacterium detected into more depth. Isolation and culturing of this bacterium and performing fermentations and characterisation of the produced mVOCs are needed to further investigate its biosynthetic capacities.

3. The hypothesis that “*Wolbachia* sp. is the strongest candidate to cause the production of characteristic mVOCs for parasitised caterpillars” (ln. 457-460) is attractive, but should be investigated in more detail to strengthen the paper. One proposed mechanism is that *Wolbachia* sp. produces the four mVOCs related to parasitism directly by itself (ln. 417-418; ln. 460). Is there any evidence (even from genome analysis) that this is possible? *Wolbachia* has an obligate intracellular lifestyle that makes it difficult to work with this bacterium to address this question experimentally. However, as the authors discuss (ln. 423-424), insect cell-lines could provide a solution to characterize the presumed *Wolbachia*’s volatiles. The other two mechanisms proposed (ln. 418-421) also deserve further experimentation.

**A:** We agree with the reviewer that further experimentation is needed to support our hypothesis that *Wolbachia* is involved in mVOC production, either directly or indirectly. So far, no data are available on mVOC production by *Wolbachia*, mainly because *Wolbachia* cannot be cultured in the laboratory, making such a study very difficult. Also for the other mechanisms proposed, indeed, further research is needed. This has now been added to the text, so that it reads as follows: “Our experiments involving micro-injections with *Wolbachia* confirm the hypothesis that *Wolbachia* is involved in hyperparasitoid host location, but further research is needed to fully understand its contribution.” It can be hypothesised that the presence of *Wolbachia* in the internal microbiome after parasitism alters the caterpillar's odour through various mechanisms, which in turn may lead to changes in hyperparasitoid behaviour. For example, changes in the caterpillar’s odour profile could be caused (i) by directly producing mVOCs which diffuse to the outside of caterpillars through the spiracles, (ii) by impacting caterpillar metabolism, thus providing other bacteria with a different set of metabolites, which are fermented to signature volatile compounds, or (iii) by indirectly affecting the bacterial communities, and thus the production of mVOCs through its presence. Its obligate intracellular lifestyle makes it difficult to cultivate the bacterium and carry out fermentations or experiments [14,20]. However, insect cell-lines could provide a solution to characterise *Wolbachia*’s volatile metabolites [60]. However, although *Wolbachia* was found to affect attraction of the hyperparasitoid B. galactopus, our results also showed that total mounting time was not affected by *Wolbachia* inoculation, suggesting that other factors are involved to fully explain hyperparasitoid behaviour.” (Lines 531-553)

**Part III – Minor Issues: Editorial and Data Presentation Modifications**

Please use this section for editorial suggestions as well as relatively minor modifications of existing data that would enhance clarity.

Reviewer #1:(No Response)

Reviewer #2:

1) The introduction includes information that occasionally appears extraneous or review-like (beyond necessary context/background). It would be helpful to make the connections to this study clearer.

**A:**We thank the reviewer for his concern regarding minor issues in our study, these contributed to increasing the quality and readability of our manuscript. We shortened the introduction by removing detailed examples in Line 71-73 and Line 80-82.

**Removed line 71-73:** Among others, recent studies have unravelled the role of their microbiome in nutrient provisioning and digestion [9,10], protection against natural enemies and pathogens [11,12] and temperature adaptation [13,14].

**Removed line 80-82:** For example, in the absence of microorganisms, human sweat and other bodily products are odourless, whereas microbial colonisation generates particular odour emission [22,23].

2) Line 75: “…Microorganisms can affect insect olfaction and behavior”

I do not disagree with this statement, however the potential for microorganisms to affect the molecular processes of olfaction is different from microorganisms’ affect on olfactory guided behavior.

**A:** Adjusted olfaction and behaviour to “olfactory guided behaviour” as suggested (Line 75).

3) Line 79: Are the parentheses necessary? “(body) odours”

**A:** We agree that the parentheses can be seen as unnecessary and removed these (Line 79).

4) Line 86-88: “Their preference is strongly linked to individuals with a higher bacterial abundance…”This is a broad statement and both supporting examples related to human skin microbiota and mosquitoes. Is there literature that supports this in relation to carnivorous/hematophagous insects in general?

**A:** Due to its importance for vector-borne disease most in-depth research linking host-microbiome and insect behaviour is aimed at human (or other mammals) and mosquitoes interaction. For other hematophagous insects and carnivorous insects, studies are often limited to testing single bacterial isolates, their fermentation products or individual compounds present in mVOCs (see Goelen et al., 2020 in *Functional Ecology*; van Neerbos et al, 2023 in *Insect Science*). These studies, among others provide plenty of evidence that the volatile products of one bacterium can affect the preference of carnivorous/hematophagous insects. Thus, we agree that the statement in line 86-88 can be considered too strong by a lack of evidence on bacterial abundance and diversity in other systems and was specified to only be known for mosquitoes. We further adjusted the sentence to highlight better that the mVOCs of single bacteria can alter insect preference.

**Line 86:** Their preference is often linked to the presence of specific microorganisms producing mVOCs [14,19,20]. For example, mosquitoes are known to be attracted towards individuals with a higher bacterial abundance and lower diversity [13,21].

5) Figure 1 – If the primary goal of figure 1 is to outline the experimental design and sampling, I do not think the description of the aluminum foil restriction device is necessary here. It was described more clearly in the methods section.

**A:** Removed the description of the restriction device in the subscript

**Removed from Line 136:** .. 12 mm sieve on an aluminium foil basis as ...

6) Line 147 – the abbreviations for experimental treatments are described here for starved (ST) and surface sterilized caterpillars (W) but the abbreviations for unparasitized (Pb) and parasitized (Cg) are not defined.

**A:** We agree with the statement that the abbreviations for unparasitized (Pb) and parasitized (Cg) treatments are not defined and added the abbreviations in the description above (L144-145).

7) Lines 161, 168, and line 7 of Table 1. Line 161 lists (Z)-3-hepten-1-ol as characteristic of parasitized caterpillars and refers to yellow highlighted lines of Table 1. Table 1 highlights (Z)-4-hepten-1-ol. Which compound name is correct? This also applies to line 168 which refers to Table A in the supporting information and line 358.

**A:** We thank the reviewer for his sharp eye regarding this important detail. The correct compound name is (*Z*)-3-hepten-1-ol. We adjusted the information accordingly throughout the manuscript and supporting tables.

8) Line 181: What is the citation [44] included in support of? Methods?

**A:** Citation 44 is indeed included in support of methods, we included a few words to clarify this.

**L181:** … as described in [44].

9) Line 182-184: It seems appropriate to put these figures (S5 Fig) in the supplementary materials, however the statistical analyses should appear alongside the interpretation.

**A:** In the revised manuscript we included the statistical analyses alongside the interpretation. Additionally, we added in the materials and method section which command was used.

**L182:** .. (Empty vs Pb-frass: p = 0.007, U = 643.5; Empty vs Cg-frass: p = 0.163, U = 565) ..

**L184:** .. (Pb-frass vs Cg-frass: p = 0.504, U = 1066.5) ..

**L731:** .. using the wilcox.test command (unpaired)

10) Table 1: The methods section indicates that these compound IDs represent tentative identification (methods lines 575-578). The figure legend should indicate that these are tentative and not authenticated identifications. Alternatively, many of these are readily available as pure standards and the IDs could be authenticated.

**A:** Added to the table legend and subscript of newly added figure 2 that compound identity represents tentative identification.

**Line 185:** … , (tentative identification)

11) Also Table 1: The figure legend (and relevant methods) indicate that compound abundance was determined by peak height rather than peak area. This seems to assume that peaks are reliably gaussian. Is there a reason to use peak height rather than peak area?

**A:** Peak area is indeed a commonly used unit for compound abundance, but we used peak height rather than peak area. As indicated in the material and method section, we used MetAlign for automated baseline correction, peak selection and alignments of all extracted mass signals of the raw data. After baseline correction, the MetAlign algorithm picks only the height of a peak and will not take overlap or peak area into account. For more detail on this, reviewers are referred to the original article on MetAlign (Lommen, 2009). This article mentions that this avoids problems with peak overlap, varying baselines, peak tailing, peak fronting, thresholds, ion suppression artifacts, and saturation artifacts and therefore miss information. We included this in the methods text to avoid any future confusion on why we peak height rather than peak area.

**Line 571:** .. . This software uses the height of the peak after baseline correction to avoid problems with peak overlap, varying baselines, and peak tailing among others [71]

12) Line 235: “parasitised, surface sterilized caterpillars (Cg-ST)”. Should this be ‘Cg-ST-W’?

Thanks for your eye for detail, this should indeed be Cg-ST-W (now CG-ST-EMD), we adjusted this accordingly.

**L235:** …(Cg-ST-EMD).

13) Line 326: “contrasting comparisons of their microbiome, odour profiles (S3 and S4 Figs) and earlier findings [44].” This reference is for a separate species of caterpillar, which should be indicated (e.g. “…earlier findings in a closely related species” or similar). It is easy to confuse this as an earlier study of the same species.

**A:** We understand that this can be confusing and applied the suggested edit.

**L326:** … and earlier findings in a closely related caterpillar species.

14) Line 378-379: “…confirming their origin and indicating that frass odour is more dependent on food plant than caterpillar species.” Presumably the two caterpillar species were reared on the same host plant? This is not indicated.

**A:** Added that both caterpillars were reared on the same plant (Brussels sprouts)

**Line 379:** … as both caterpillars were reared on Brussels sprouts plants.

15) Lines 384-386: Please make it clear that being a known/possible mVOC does not mean that a compound cannot appear in non-microbial contexts. Many of the compounds discussed here are reported in a variety of taxa.

**A:** Previous wording could be interpreted as misleading, we now briefly mention that these VOCs are also present in other taxa.

**L383:** Among their presence in various taxa, three out of four characteristic VOCS for parasitised caterpillars are known to be mVOCs.

16) Line 663: Permanova analyses are frequently accompanied by analyses of dispersion, as permanova alone does not distinguish between differences in centroid versus dispersion. Were tests done to distinguish between differences between multivariate means/centroids and differences in spread/variance?

**A:** The reason why we did not include this analysis, is that we were mainly interested in differences in means and not in variances. Moreover the function (betadisper) does not work when you have several factors (as we had).

17) Line 1039: Mann-Withney U-test should be Mann-Whitney.

**A:** Adjusted the spelling of “Withney” to “Whitney”.

Reviewer #3:

1. The indistinct use of the words "microbiome" and "microbiota" is incorrect. See the sentence in line 65: “The microorganismal part of the holobiont also called microbiome or microbiota”, but also check throughout the text.

**A:** Thanks for your comment and eye for detail. Microbiota is indeed incorrect in this context and was adjusted to microbiome in line 65 and 597.

2. Some results seem to partially inconsistent with those previously published, and the inconsistences are not sufficiently discussed. See the title of the ref. 47: “Parasitism by endoparasitoid wasps alters the internal but not the external microbiome in host caterpillars.”, in contrast with what is reported in the abstract of the present paper “We found that parasitism by *C. glomerata* … significantly affected the internal and external microbiome of the caterpillar, which were both found to have a significant correlation with caterpillar odours”, and also ln. 245-252.

**A:** Thank you for pointing out these inconsistencies. We incorporated the inconsistencies with ref 47 better in a new paragraph in the discussion and note that i) the external microbiome is altered in our current study (and is not in [47; Gloder et al., 2021]); ii) the Shannon diversity is affected in a different way. We briefly discuss the different circumstances in the studies leading up to these differences and how our study has a more robust design, allowing us to find more subtle differences. Furthermore we sharpened the presentation of our findings on the Shannon diversity, to better reflect these results.

**Line 248-250:** The diversity of the external bacterial community decreased after parasitism and the diversity of the internal bacterial community increased. Treatment of caterpillars reduced the diversity of the external, but not the internal microbiome ….

**Line 451:** We found that both the external and internal microbiome of *P. brassicae* caterpillars are altered after parasitism by *C. glomerata*. In contrast, our prior study indicated that C. glomerata parasitism only affected the internal microbiome of *P. brassicae* [47]. Furthermore, in our previous study we found a reduction in the diversity of bacterial communities for both internal and external microbiomes after parasitism, whereas our new data identified relatively weak effects. Both studies are on the same study system, but previous findings [7,63–65] suggest that habitat is one of the strongest factors influencing the external microbiome of insects, outweighing the effects of parasitism due to differences in day, weather, environment and/or host plant [41]. As a result, parasitism status played an insignificant role in explaining variation in the external microbiome. To further investigate differences caused by parasitism, our current study pooled the microbiomes of groups of caterpillars and only examined one habitat (lab strain). This allowed us to identify that the external microbiome is altered alongside the internal microbiome. The difference in responses of Shannon diversity can most likely be attributed to the studied lab strain with an overall less diverse bacterial community [41] and to the higher resolution with more statistical power in this study.
